# Mental health challenges experienced by LGBTI+ community in Gaborone: A phenomenological study

**DOI:** 10.4102/hsag.v28i0.2347

**Published:** 2023-09-29

**Authors:** D. S Mangwegape, Eva Manyedi, Boitumelo J. Molato

**Affiliations:** 1 Department of Psychiatric Mental Health Nursing, Faculty of Nursing, Institute of Health Sciences, Lobatse, Botswana; 2 Department of Nursing, Faculty of Health Science, North West University, Mahikeng, South Africa

**Keywords:** challenges, depression, gender dysphoria, LGBTI+, loneliness, isolation, mental health

## Abstract

**Background:**

Mental health challenges have affected the entire global population including individuals identifying as lesbian, gay, bisexual, transgender, intersex and others (LGBTI+). There is documented evidence of a high prevalence of mental health challenges among LGBTI+ community across the globe, but in Botswana there is dearth of literature pertaining to the phenomenon of LGBTI+ mental health challenges.

**Aim:**

The study was aimed at exploring and describing the mental health challenges experienced by some people identifying as the LGBTI+ community in Gaborone, Botswana.

**Setting:**

The study was conducted in Gaborone in Botswana.

**Methods:**

The study adopted a qualitative, phenomenological, descriptive design with 15 participants identified through snowball sampling. LEGABIBO, the LGBTI+ advocacy organisation, served as gatekeeper after Health Research and Development Division under the Ministry of Health and Wellness gave the ethical clearance of the study. Data were collected through unstructured telephonic interviews and recorded with a digital voice recorder.

**Results:**

The study established that some LGBTI+ individuals experienced mental health challenges like experiences of depression, experiences of gender dysphoria, and loneliness and isolation.

**Conclusion:**

It is concluded that individuals identifying as LGBTI+ experience mental health challenges that stem from being stigmatised and discriminated among others.

**Contribution:**

The findings of the study provide information that may be used in dealing with mental health issues of individuals identifying as LGBTI+. Furthermore, the findings may inform nursing practice, research and education issues on LGBTI+ as well as influence health policy in addressing the mental health issues of those identifying as LGBTI+.

## Introduction

There are so many diverse groups within the general population with some classified on the basis of sexual orientation (Moleiro & Pinto 2015:1). One such grouping which is in the minority, is the lesbians, gays, bisexual, transgender, intersex, and other gender diverse individuals (denoted +) collectively known under the acronym LGBTI+ (Lee, Ylioja & Lackey 2016). There are a variety of other acronyms (gays, lesbian and bisexuals [GLB], gays, lesbian, bisexual, transgender, intersex and queer [GLBTIQ], and lesbian, gays, bisexual, transgender, queer and asexual [LGBTQA]) that denote several of these group of people (Moagi et al. 2021:^1^). In the context of this article, LGBTI+ will be used to signify all individuals identifying within sexual orientation and gender identity (SOGI) minority community.

Those identifying as lesbian, gay, bisexual, transgender, intersex and others (LGBTI+) usually experience stigmatisation and discrimination at individual, institutional and structural level (Duhaylungsod et al. 2018:1; Muller 2017:2). According to Russell and Fish (2016:13), discrimination of individuals identifying as LGBTI+ came into the spotlight in the 1960s when ‘homosexuals’ in the United States (US) were classified as a ‘sociopathic personality disturbance’ by the American Psychiatric Association (APA) through the Diagnostic Statistical Manual of Mental Disorder (DSM)-2nd version (DSM-II). Despite this classification being removed from both the International Classification of Disorders (ICD-9) and DSM-II, its legacy still propagates stereotypes and prejudice on LGBTI+ groupings which leads to adverse mental health outcomes (Spurlin 2019:10–12).

The removal of homosexuality as a disorder is not the only effort to address LGBTI+ issues as same-sex marriages have been legalised in several countries like the US, Ireland, Australia, and South Africa (Healy, Thomas & Pedersen 2015:389; Yarbough 2018:1093).

Documented evidence is abounding that individuals identifying as LGBTI+ face health problems on account of stigma and discrimination, including mental health problems (Cyrus 2017:194). Kneale and Becares (2021:1) further assert that discrimination explains the high levels of poor mental health among individuals identifying as transgender. As observed by Acharya et al. (2017), the LGBTI+ community suffer from mental health problems that include anxiety disorders, depression, and problematic substance use. Mental health is significant for those identifying as LGBTI+ as it ensures that they enjoy healthy relationships and fulfilment of life (Moagi et al. 2021:2).

There is reported evidence of mental health challenges in the African context as well. Men who have sex with men (MSM) in Nigeria had minority stress which impacted on the participants’ mental health (Makanjuola, Folayan & Oginni 2018:379). Furthermore, a study in Kenya, Lesotho, Swaziland, and South Africa found that the research participants displayed depression and anxiety symptoms while others were dependent on alcohol and have made suicide attempts (Muller & Daskilewicz 2018: 271).

In Botswana, studies conducted among LGBTI+ community were mostly to deal with key population interventions of HIV and/or AIDS (Mashumba 2019:161). Only a few studies have been conducted to explore this phenomenon. One study investigated the wellbeing of lesbian, gays and bisexuals (LGB) with suicidal ideation and emotional distress being the mental health challenges experienced by the study population (Ehlers, Zuyderduin & Oosthuizen 2001:848–855; Selemogwe & White 2013:409–410). The study was, however, concentrated only on few of the groupings of gays, lesbians and bisexuals within the LGBTI+ community. The study was also directed in an era when the country was highlighted to be repressive in attitudes towards LGBT (BONELA et al. 2008:2; Tabengwa & Nicol 2013:340).

With this being said, the researchers saw a need to conduct the empirical study about mental health challenges experienced by LGBTI+ community in Botswana. In addition, this phenomenon of mental health challenges among LGBTI+ community was also evidenced by the first author during the years of practice in a mental health care institution. The researcher assisted clients with bouts of depression and suicidal ideations after being rejected by their families following the process of ‘coming out’. As postulated by Hall (2018:1), parental rejection is one of the prominent risk factors for depression of LGB individuals.

Based on the above, an exploratory study is needed to explore mental health challenges experienced by the LGBTI+ community in Gaborone, Botswana.

## Aim

The study was done to explore and describe mental health challenges of the LGBTI+ community in Gaborone, Botswana.

## Research methods and design

### Study design

The study adopted a qualitative, descriptive, phenomenological design. Because the study was aimed at exploring mental health challenges from the lived experiences of LGBTI+ community, the design was appropriate (Polit & Beck 2022:165).

### Setting

The study was conducted in Gaborone, the capital city of Botswana, located in the southern part of Botswana. Gaborone has an extensive network of public and private health facilities that include a national referral hospital and three private hospitals as well as clinics. It has an estimated population of 289 703 (Statistics Botswana 2022:5). Gaborone is a cosmopolitan city and has people from diverse cultural groups with English and Setswana being the main languages.

### Study population and sampling strategy

The population for this study was people identifying themselves as LGBTI+. In selecting research participants, snowball sampling method was used. Snowball sampling is a method whereby research participants make referrals for others to be research participants and is often used with minority or hard to reach populations (Polit & Beck 2022:176). Initial participant was established through gatekeepers, LEGABIBO. The rest of the participants were identified by the participants themselves. The sample population was 15 participants who were arrived at after data saturation. Data saturation was achieved at 12 participants and 3 more participants were interviewed to confirm the same.

Inclusion criteria involved individuals self-identifying as LGBTI+, had attained 18 years of age and above, had disclosed their identity to the advocacy organisation, voluntarily participated in the process, communicated in English and/or Setswana, and resided in Gaborone. Individuals who were not self-identifying as LGBTI+ and were below 18 years were not included in the study. Furthermore, individuals who were not residing in Gaborone and were not citizens of Botswana were also excluded.

### Data collection

Data were collected from June 2021 to August 2021 through unstructured individual interviews with an interview guide. The interview guide was developed in English and Setswana. The interviews which lasted for approximately 60 min, started with the following open-ended question being posed to the participants: ‘Tell me about the mental health challenges if any, that you experience as an LGBTI+ individual?’ was asked. To further facilitate the discussion, communication techniques like clarifying, reflecting, and summarising were used by the researcher.

Data which were collected telephonically to minimise coronavirus disease 2019 (COVID-19) transmission were recorded by a voice recorder. Each interview was given a code and data were then safely kept in a computer which was password-protected. The data were transcribed verbatim by the researcher following the telephonic interviews that were audio-recorded.

### Data analysis

Data were analysed by the researcher and an independent co-coder guided by a modified Collaizi’s data analysis method (Finlayson et al. 2018:3). The method is used to analyse phenomenological data, hence suitable for this study.

The data analysis process followed several procedural steps that include describing the phenomenon of the study, collecting participants’ descriptions of the phenomenon, reading all participants’ descriptions of the phenomenon, returning the original transcripts and extracting significant statements, spelling out the meaning of each significant statement, organising and then aggregating the formalised meanings into clusters of themes (Finlayson et al. 2018:3). Lastly, a comprehensive description of codes was written and sent to participants for validation of the description. There was no new data following validation which led to the researcher and independent co-coder cordially agreeing on the themes that were presented in narrative and descriptive format.

### Measures to ensure trustworthiness

Trustworthiness is the gauge through which the research quality is measured (Bradshaw et al. 2017:5). Several strategies were enforced to ensure the quality using Lincoln and Guba’s framework (Polit & Beck 2022:277). In ensuring trustworthiness, several strategies were employed that include: credibility, transferability, dependability, confirmability, and transparency.

In ensuring transparency, the recruitment procedure for research participants and the data analysis process have been clearly outlined in the report. Direct quotations of research participants have been used in the report to enhance confirmability of the data. Raw data like field notes and verbatim transcriptions were availed to ensure confirmability. An audit trail of what happened in systematic data collection and data analysis process is available; a process that will highlight the dependability of the research.

In order to ensure transferability, data interpretation was supported by evidence-based literature. In ensuring credibility, the telephonic interviews were audio-recorded and then transcribed verbatim with participants given an opportunity to confirm the findings. Data saturation was reached after 10 interviews, with 5 more interviews conducted to guarantee rich data. There was prolonged engagement with the participants, and also involvement of an independent co-coder in data analysis to safeguard the veracity of findings.

The researcher kept a reflective journal which encompassed the researcher’s personal reflections. A reflexive journal was also kept to avoid the researcher being expressively immersed with the participants which could have contaminated the data (Polit & Beck 2022: 279).

### Ethical considerations

The ethical clearance to conduct the study was granted by the Ministry of Health and Wellness through the Health Research and Development Division (HPDME 13/18/1) for the study to be conducted in Botswana. Goodwill permission was offered by the advocacy organisation, LEGABIBO. An independent person informed the participants on their right to self-determine their participation or withdrawal from the study. Identities of the participants were concealed and were only identified as Participant 1, 2, 3 and so on during the data selection process. The participants were not coerced to participate in the study, and they were informed about their rights to stop participating anytime when they feel so.

## Results

Three themes and three sub-themes emerged from this study (see Table 1 for more details).

**TABLE 1:** Categories and sub-categories.

Theme	Sub-theme
1. Experiences of depression	1.1 Suicide attempts
	1.2 Low self-esteem
	1.3 Not being fully themselves
2. Experiences of gender dysphoria	-
3. Loneliness and isolation	-

*Source*: Mangwegape, D.S.M., 2022, ‘Mental health challenges among the Lesbian, Gay, Bisexual,Transgender and Intersex community in Gaborone, Botswana’, Master’s Thesis. North-West University.

### Theme 1: Experiences of depression

Three sub-themes identified under theme 1 were namely, suicide attempts, low self-esteem, and not being fully themselves.

‘Experiences of depression’ emerged as the first theme following data analysis. The participants self-reported depression that was validated by various emotions that included expressions of worry, frustration, stress and sadness, and below are some of their expressions:

‘And I started having too much anxiety and depression because I was asking myself on what if after lockdown I am going to go into the society and look very different from how I was before lockdown commenced. It was very depressing.’ (Participant 4, Transgender woman, Tertiary Student)

Another participant echoed the same sentiment:

‘That also contributed to my depression because after that I felt unsafe at all.’ (Participant 8, Transgender woman, tertiary student)

Another participant added by saying that:

‘When I am a bit depressed, I wouldn’t want people to know. I am always laughing even though I am not okay.’ (Participant 9, Transgender man, volunteer)

Some participants expressed that COVID-19 was a factor that contributed to them experiencing depression. The discussion unfolded as follows:

‘In the advent of corona in 2020, I experienced challenges since I was staying alone without any one to offer comfort and support and ended up having depression which nearly disabled me.’ (Participant 1, Gender non-conforming, Hair dresser)

#### Theme 1.1: Suicide attempts

The research participants shared their versions of having attempted suicide on the basis of discriminating tendencies by the community. Below are some of their expressions:

‘I wanted to commit suicide. The question was why this was happening to me God, why is the community shooting me like this, where I should go. Why me?’ (Participant 1, Gender non-conforming, Hair dresser)

Another participant added by saying that:

‘This was either I die or stop living.’ (Participant 11, Transgender woman, LGBTI+ activist)

One of the participants engaged in substance use to try to take his life; fortunately, he recovered after been admitted at the hospital. This was expressed as follows:

‘Time of the incident I was doing Form 3, my mom and dad were undergoing divorce. I felt that because I am their child, they are doing it because of me. Because I realised that I was the only one with disability issues. I ended up feeling that I should get away from them. I drank several substances and was admitted but managed to recover.’ (Participant 13, Intersex, Unemployed)

Family rejection was attributed by one of the participants as a factor increasing vulnerability to suicide and had this to say:

‘Saying from personal experience I have tried to commit suicide three times thank God because it failed. Being me in this society is something like you are an outcast or something that is unusual by the society.’ (Participant 10, Transgender man, LGBTI+ Activist)

#### Sub-theme 1.2: Low self-esteem

Having low self-esteem was expressed as impacting negatively on the mental health of several research participants. Inability to do gender affirmation surgery (GAS), being portrayed differently from their gender was ascribed by participants as leading to low self-esteem. One of the participants expressed this as follows:

‘Make you to look down upon yourself and see yourself as worthless.’ (Participant 5, Bisexual, Teacher)

The same sentiment was shared by another participant who responded by saying that:

‘Some of the things is low self-esteem, which other people don’t experience.’ (Participant 9, Transgender man, volunteer)

Another participant expressed:

‘At senior school all was fine, but I found myself having low self-esteem because of ball sports.’ (Participant 14, Intersex, Unemployed)

Participants also indicated that their low self-esteem was aggravated by being questioned on their LGBTI+ status and stigma. One of the participant stated:

‘I ask myself that is it really the right discovered life that I should be living as in like is being gay okay, is being who I am okay, maybe I am doing something wrong and again get to a point of questioning and say if I say or they say I am wrong why didn’t God make me normal like they say?’ (Participant 3, Gay, Health professional)

#### Sub-theme 1.3: Not being fully themselves

The sub-theme of LGBTI+ not fully being themselves is related to the major theme of experiences of depression. As deduced from field notes, research participants shared feelings of disappointment and dejection and thus succumbing to societal pressures of not being themselves. One participant had this to say:

‘I wanted to identify as transwoman, but because of the circumstances in my life I couldn’t be the full woman I wanted to become thus I chose to be gender non-conforming minority group within the LGBTI community.’ (Participant 1, Gender non-conforming, Hair dresser)

Another participant expressed:

‘This idea, whole attitude or triggers of how people react when they see us, it got me to a point where I ended up having to change to the person I am right now.’ (Participant 3, Gay, Health professional)

Another participant shared views on not being able to be involved in relationships that were fulfilling for them:

‘I fell for a girl but I ignored the feeling because of what society and my parents will say as they were openly critical of that.’ (Participant 5, Bisexual, Teacher)

Another participant echoed the same views:

‘I have always not fully displayed my affection with partner publicly; in other words, I respect the community.’ (Participant 15, Lesbian, Bar tender)

### Theme 2: Experiences of gender dysphoria

The findings revealed that some of the participants identifying as LGBTI+ experience gender dysphoria (GD). The participants expressed having feelings of some incongruence with their bodies on several occasions. One of the participants stated:

‘The way you appear and the way you feel do not match. It is stressing … It is like a fight between your mind and body.’ (Participant 6, Lesbian, Waitress)

Another participant expressed feelings of being trapped in the wrong body and said the following;

‘The biggest challenge before transition was that I was born in the wrong body.’ (Participant 7, Transgender man, Store assistant)

Another participant echoed similar views as follows:

‘It’s a feeling that I am born in the wrong body.’ (Participant 11, Transgender woman, LGBTI+ activist)

Majority of the participants who were diagnosed with GD also had anxiety and depression. One of the participants had this to say:

‘The condition that I told you about earlier on is called gender dysphoria. It results in me being depressed and having a bit of anxiety and I had to be given antidepressants to avoid situations where I become suicidal because of the way I am.’ (Participant 4, Transgender woman, Tertiary Student)

### Theme 3: Loneliness and isolation

The last theme that emerged was loneliness and isolation. Several of the participants expressed finding it difficult to interrelate with people because of the feelings of inadequacies in them as well as of not belonging. It was apparent from some of the research participants’ perceived frustration that this was a problem which led to their isolation.

Participant 1 had this to say:

‘Felt lonely, I felt I wanted to be loved; I felt left out.’ (Participant 1, Gender non-conforming, Hair dresser)

Another participant who shared the same view added by saying that:

‘When I get home, I get to discover that I am alone.’ (Participant 2, Gender non-conforming, Model)

The following was echoed by another participant:

‘I didn’t want to socialise, I didn’t want to do nothing, I didn’t want to participate in anything either in family gatherings or picnics. I will just be always home.’ (Participant 8, Transgender woman, Tertiary student)

Another participant who accepted himself expressed as follows:

‘It is a personal experience, I am okay on my own because I don’t wanna be questioned, I don’t wanna be ridiculed, and I don’t wanna be finger pointed at.’ (Participant 10, Transgender man, LGBTI+ Activist)

A participant who could not accept their gender echoed the following:

‘The thing is when I was with the other boys, I did not feel that I am part of the crew because when it came to some conversations I will keep shut, I can’t comment.’ (Participant 12, Intersex, Unemployed)

## Discussion

The study examined the mental health challenges endured by some members of the LGBTI+ community. Three themes emerged from the study namely, experiences of depression, experiences of GD, and loneliness and isolation.

### Experiences of depression

Experiences of depression was the first theme of the study as most of the participants expressed having feelings of depression. The findings of the study are consistent with a systematic review of studies which found that LGBTI+ community are increasingly vulnerable to depression and suicide (Mongelli et al. 2019:47; Harper et al. 2021:9). Furthermore, some studies conducted in Africa, notably in Kenya and South Africa, had participants highlighting to have experienced depression (Harper et al. 2021:9; Muller & Daskilewicz 2018:271).

Some research participants had depression compounded by COVID-19. Suen, Chan and Wong (2020) posit that the advent of COVID-19 resulted in a significant number of LGB individuals experiencing clinical depression and generalised anxiety disorder. The impact of COVID-19 was also observed in a study in Australia as gender and bisexual men had increased rates of depression and anxiety (Bavinton et al. 2022). Elsewhere in Canada during the pandemic, LGBTI+ individuals were diagnosed with moderate to severe symptoms of depression (Abramovich et al. 2021:8).

Suicide attempts was the first sub-theme in this study. In the current study, participants who identified as transgender reported having attempted suicide. Existing literature highlights that there is increased vulnerability to suicide among the LGBTI+ community, with the act of discriminating and victimising transgender individuals increasing their risk for suicide (Rood et al. 2015:272). Previous studies have also highlighted increased suicidal ideations, thoughts and attempts in South Africa, Kenya, and Botswana (Ehlers et al. 2001:848; Muller & Daskilewicz 2018:271).

Another sub-theme was the experience of low self-esteem among individuals identifying as LGBTI+. Most of the participants in the current study underlined that their self-esteem was dented by issues of homophobia and transphobia which is evident in other studies also (Taylor et al. 2019:19). There is however, contrasting findings as a study in Brazil found that high rates of self-esteem in a majority of the participants (Canali et al. 2014:4509). In the aforementioned study, the participants who were graded low in self-esteem also had additional adverse factors other than gender minority status (Canali et al. 2014:4509).

The last sub-theme is that of LGBTI+ individuals ‘not being fully themselves’. There is documented evidence that inability for LGBTI+ individuals to express who they are can lead to adverse mental health problems with generalised anxiety and depression being the notable disorders (Feinstein et al. 2020:8; Rood et al. 2017:710). The research participants concealed their identity and could not express themselves when interacting with the general community, mainly out of fear of being adjudged by members of the community. Transgender individuals often conceal their gender-identity in community and/or social circles as a way of evading stigma and discrimination (Rood et al. 2017:8). Moreover, concealment of status as transgender or gender suppression, has been documented to decrease sense of belonging as well as adverse mental health problems (Brady et al. 2022). Low self-esteem is aggravated by several minority stressors that include fear of being victimised and concealment of gender-identity (Bridge, Smith & Rimes 2019:4).

### Experiences of gender dysphoria

Experiences of GD emerged as another challenge experienced by some of those identifying as transgender and to some extent, intersex. Gender dysphoria is a term to describe the mismatch between the gender expressed and/or experienced by a person and the one assigned at birth (Spurlin 2019:11; Townsend & Morgan 2018:646). Furthermore, for a clear diagnosis of GD to be confirmed, individuals should present with distress regarding the incongruence in schools, occupation and in social circles for a period of 6 months or more. For a tentative diagnosis of GD to be determined, transgender individuals must experience substantial emotional discomfort (Hall, Mitchell & Sachdeva 2021:7).

Identifying as transgender is itself not a mental health challenge, but rather a problem arises when the gender identity causes the emotional distress or discomfort (Stroumsa 2014:e31). Not every research participant who identified as transgender experienced GD as it is not always that individuals identifying as transgender will have a diagnosis of GD (Byne et al. 2018:60).

Some participants mentioned having experiences of GD without a mental health professional validation, whilst others had the diagnosis following a mental health assessment by a psychiatrist. Gender dysphoria includes a number of aspects ranging from a feeling to a diagnosis which means that GD can be either a diagnosis or a symptom (Byne et al. 2018; Lindley & Galupo 2020:6).

Some participants mentioned having GD occurring concurrently with other mental disorders. Freitas et al. (2019:101) posit that there is a high risk of mental disorders like depression and anxiety among transgender individuals. Moreover, low self-esteem, dissatisfaction with body and poorer social functioning also occur concurrently with GD (Witcomb et al. 2017).

### Loneliness and isolation

The last theme of this study was loneliness and isolation. Loneliness is the perception of lacking connection with others, whereas isolation is the nonexistence of a meaningful connection with other human beings (Gardiner et al. 2018:148). Loneliness is deemed as a health problem as most participants identified it as a challenge, which is consistent with a study by Gorczynski and Fasoli (2022:112). Participants expressed that they do isolate themselves because they do not belong and avoid being questioned. The findings are consistent with those from several studies which observed that those identifying as LGBTI+ are at a higher risk for social isolation when they conceal their identification as ‘LGBTI+’ and yield to societal prejudice which leads to internalisation of negative emotions (Perone, Ingersoll-Dayton & Watkins-Dukhie 2020:12). Andersen et al. (2020:4) further contend that individuals identifying as transgender had high incidences of loneliness in comparison to cisgender individuals.

A review by Zeeman and Aranda (2020:10) highlighted that people with intersex variations have increased feelings of social isolation and anxiety. Some participants identifying as intersex did isolate themselves on account of ‘not wanting to be discovered’. Individuals identifying as intersex do not reveal much about themselves and consequently often isolate themselves (Henningham & Jones 2021:600). Furthermore, social isolation may also be a ‘self-preservation’ tool to avoid hostile reactions from members of the public.

### Limitations

In order to mitigate risks of COVID-19 and to conform to the regulations at the time, during data collection, interviews were modified from qualitative face-to face interviews to telephonic interviews. It was difficult to observe non-verbal cues during telephonic interviews which limited the field notes. Given the nature of the phenomenon being investigated, observations of participants’ behaviour and emotional reactions would have helped generate rich results.

Despite the various members of the LGBTI+ groupings having universal commonalities, significant differences do exist also. This was another limitation of the study as the varied differences complicated the data analysis process. Consequently, an in-depth exploration of mental health challenges within particular groupings would have yielded rich data.

### Recommendations

The study recommends further research on the strategies to deal with mental health among individuals identifying as LGBTI+. The sub-groupings of intersex and transgender appear to be more vulnerable to mental health challenges, hence a study particularly focused on the specific groupings is recommended.

## Conclusion

The findings provide a basis to conclude that some members of the LGBTI+ community experience mental health challenges. The study identified that some members of LGBTI+ community experience depression and there may be suicidal ideations, poor self-esteem, and not being fully themselves largely because of non-acceptance. It is evident that GD is another challenge that is mostly experienced by those identifying as transgender and intersex. Individuals identifying as LGBTI+ on account of prejudices, stigma, and discrimination experience loneliness and isolation.
